# Generating and Modeling Virtual Patient Data from Published Population Pharmacokinetic Analyses: A Vancomycin Case Study

**DOI:** 10.3390/ph18111748

**Published:** 2025-11-17

**Authors:** Moeko Suzuki, Hidefumi Kasai, Takahiko Aoyama, Yasuhiro Tsuji

**Affiliations:** 1 Department of Practical Pharmacy, Nihon Pharmaceutical University, Saitama 362-0806, Saitama, Japan; 2 Laboratory of Clinical Pharmacometrics, School of Pharmacy, Nihon University, Funabashi 274-8555, Chiba, Japan; 3 Laboratory of Pharmacometrics and Systems Pharmacology, Keio Frontier Research and Education Collaboration Square (K-FRECS) at Tonomachi, Keio University, Kawasaki 210-0821, Kanagawa, Japan

**Keywords:** meta-analysis, population pharmacokinetic model, vancomycin, therapeutic drug monitoring, clinical pharmacometrics

## Abstract

**Background/Objectives**: In recent years, clinical pharmacometrics has become vital for drug development and clinical practice, particularly for predicting drug efficacy and safety. Population pharmacokinetic models are used for drugs for which therapeutic drug monitoring is recommended in clinical practice. Numerous population pharmacokinetic models have been developed for patients with similar clinical and demographic characteristics, resulting in reduced inter-individual variability. Despite the existence of diverse-population pharmacokinetic models, selecting an appropriate model for bedside use remains challenging. This study proposes a model-simulated model-based meta-analysis (M-cubed) to construct a unified model capable of accommodating a wide range of patient backgrounds. **Methods**: Vancomycin (VCM), a drug used for therapeutic drug monitoring, was used as an example. Using information from published VCM models, the M-cubed method was employed to generate virtual patient data for each publication through simulation, followed by modeling the integrated dataset. **Results**: Population pharmacokinetic analysis was performed on data from 19 virtual patient models, resulting in a total of 2303 cases. Covariates in the final model included creatinine clearance and body weight. The predictive ability of the model was robust. **Conclusions**: A model that integrates several population studies using the M-cubed method is required to address the need in clinical practice.

## 1. Introduction

In clinical trials for pharmaceutical development, clinical pharmacometrics is commonly used to explore dosage regimens for ensuring efficacy and safety. Ideally, clinical trial subjects should encompass as broad a range of backgrounds as possible to reflect postmarketing use. However, patients with special backgrounds or diseases are typically excluded during the drug development phase; nonetheless, in real-world clinical settings, drugs may need to be administered to types of patients that have been excluded from clinical trials. Vancomycin (VCM) is a representative agent used to treat Gram-positive bacterial infections such as those caused by methicillin-resistant *Staphylococcus aureus* (MRSA) [1]. In clinical trials of VCM, studies on patients infected with MRSA who exhibited special pharmacokinetic (PK) behavior (e.g., patients with cancer or burns) were excluded [2]. However, patients with severe injuries and a high risk of infection are precisely those who require aggressive treatment.

VCM is commonly used for the treatment of infectious diseases worldwide. In Japan, the precise dosage regimen for VCM relies primarily on population parameters reported in 1998 [3,4].

The covariates in the population pharmacokinetic (popPK) model of clearance (CL) include only creatinine clearance (CLcr). If clinical pharmacists consider dose recommendations for patients whose PKs are influenced by factors other than CLcr, the predictions of individual patient PK parameters and blood drug concentrations proposed by the model may be unreliable, also considering that the patient data used to build the popPK model may not necessarily include special patient groups such as those with augmented renal clearance [2]. In clinical settings, some patients present challenges in recommended dosing in existing applications. The population model [3] incorporated into the existing VCM dosing application [4] does not include data from patients from so-called special populations. Therefore, the dosing regimens recommended by this model may not be suitable for the treatment of patients with atypical PKs.

As of December 2024, over 350 popPK models for VCM were available. Three major problems are associated with the large number of reports. First, the numbers of cases and blood concentration points were inadequate for modeling papers without prior consideration of the sampling design. This is mainly because most studies were retrospective and used therapeutic drug monitoring (TDM) data from clinical practice. Second, the existence of hundreds of models complicates the selection of the most suitable model for individualized dosing design. Third, although hundreds of models have been reported, many may have never been utilized. Regardless of the number of models available, a fundamental knowledge of pharmacometrics is essential for pharmacists to personalize appropriate dosage regimens.

These issues can be addressed if the popPK model [3] integrated into the application [4] can be applied to dosing optimization for all patient backgrounds; however, recruiting a sufficient number of patients across a wide range of diseases is challenging, which in turn limits the potential to conduct large-scale studies that can capture the variability in PK parameters. Therefore, we employed meta-analysis to integrate multiple models encompassing various patient backgrounds.

**Figure 1:**
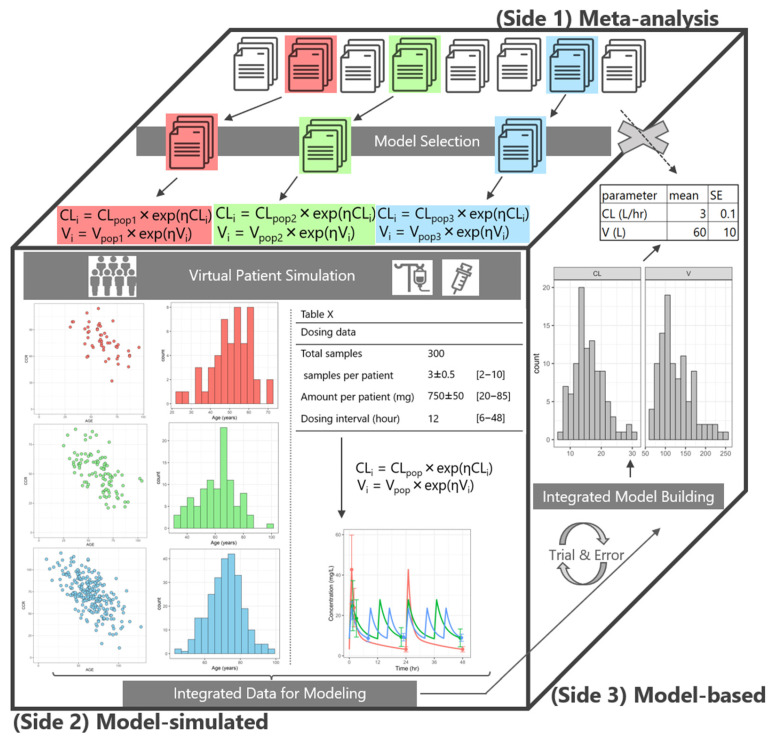
This figure illustrates the fundamental components of the model-simulated model-based meta-analysis (M-cubed) method divided into three sides. **[M-cube side 1: Selection of Population PK Models]** First, appropriate population pharmacokinetic (popPK) models (research papers) are selected for integration. Notably, unlike conventional meta-analysis, it is impossible to integrate population models using only published literature information. This is because the publications reporting these models typically present only the model analysis results (parameter estimates), and the reported models are not often differed in terms of either the structural model or the covariate model. (A gray cross mark between Side 1 and Side 3.) **[M-cube side 2: Generation of Simulated Data]** To obtain the raw data required for model building, virtual patients are generated. First, virtual patient profiles (demographic data), such as number of subjects, age, weight, and renal function, are generated for each study based on the summary statistics of the original patient characteristics reported in each publication. Next, simulated drug concentrations are generated using the popPK model from the respective study. During this process, the simulation faithfully reflects the reported dosing regimen (dose, dosing frequency), and the number and timing of sampling points. This step is repeated numerous (typically 200 to 1000) times. **[M-cube side 3: Integrated Population PK Modeling]** Finally, the integrated popPK model is constructed using the generated virtual data. Specifically, a single integrated dataset is created by pooling all virtual patient data generated in Side 2, and modeling is performed on this consolidated data. This model building process is repeated for each iteration of the multiple simulations (virtual data generation). Ultimately, the population parameter estimates obtained through these numerous modeling attempts are aggregated and evaluated by summarizing their mean values and their standard errors.

To the best of our knowledge, no methodology for integrating popPK models has been reported to date. Model-based meta-analysis (MBMA) has been widely used, particularly in pharmaceutical development [5,6]. However, MBMA cannot be used to merge mathematical models themselves. On the other hand, methods for precise dosing for individual patients have been proposed, such as machine-learning (ML)-based selection of the optimal model [7] and VCM’s model-informed precision dosing (MIPD) approach [8,9,10]. However, these approaches all involve selecting from a set of existing models and thus have the limitation of not being able to accommodate a broad range of patients. Therefore, the objective of the study was to develop a novel methodology, the model-simulated model-based, meta-analysis (M-cubed) method (Figure 1). This methodology aimed to construct popPK models using simulated and integrated patient data from multiple studies. The meta-analysis integrated results from tables and figures of each article. The model, developed using the M-cubed method, is a theoretical mathematical model that is identical to one constructed using conventional population analysis. The M-cubed method specifically integrated reported popPK models. By extending the methodology of meta-analysis and using simulation techniques, our approach facilitated the integration of several mechanistic models across various patient backgrounds. We then tested whether multiple popPK models for clinical use could be integrated using the M-cubed method. We thus addressed the need for individualized dosing strategies for patients with atypical PK profiles in clinical settings.

## 2. Results

### 2.1. M-Cube Side 1

**Figure 2:**
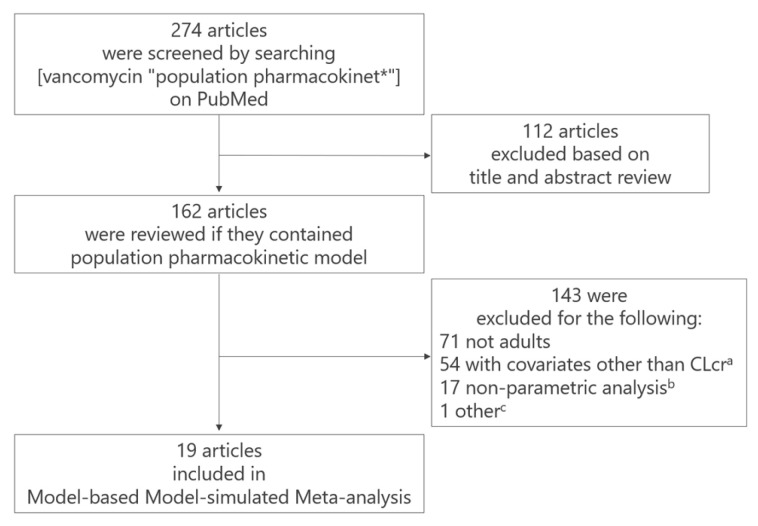
Flowchart of study selection. As of September 2022, 698 articles were identified, of which 19 studies of popPK analysis of vancomycin in adults were included.

A flowchart of the study selection process is shown in Figure 2. We targeted VCM, because of the existence of a relatively large number of popPK model reports. Nineteen selected models were integrated, and 19 studies had been conducted on adult patients. These models included only CLcr in the CL model. Of the 19 models, three were popPK analyses of VCM in Japanese patients. Table 1 summarizes the patient backgrounds and characteristics of the 19 models. The number of patients in these studies ranged from 14 to 398. Overall, many studies included a higher proportion of male than female participants. In addition to general infection patients, the patient background in the studies included those undergoing allogeneic hematopoietic stem cell transplantation, patients with obesity or hematological malignancies, critically ill patients with sepsis or external ventricular drain-associated ventriculitis, patients receiving extracorporeal membrane oxygenation therapy, patients with burns, and those with post-craniotomy meningitis.

The 19 population CL parameters are summarized in Table S1. Ten, eight, and one studies used one, two, and three-compartment models, respectively. The CL formula comprised 11 linear and eight power models. For models where residual unidentified variability was reported as σ^2^ in nonlinear mixed-effects model analysis, σ^2^ values are listed in Table S1.

(a) 54 studies were excluded because their CL models included covariates other than creatinine clearance (CLcr) estimated using the Cockcroft–Gault equation [11]. Although the Cockcroft–Gault equation for CLcr is common in clinical settings worldwide, estimated glomerular filtration rate (eGFR) formulas (e.g., [12,13]) vary among studies, making standardization impossible. This is the reason why we included models which had CLcr as a covariate.
(b) 17 studies were excluded due to the use of nonparametric methods,
(c) one article was excluded because the reported estimates of clearance differed greatly from those reported in other studies due to unknown reasons.

**Table 1:** Patient background and characteristics of the 19 models selected using the M-cubed method.

Study		Number of Patients	Purpose of the popPK Model	Characteristics ^a–d^				Reference
No.	1st Author (year)			Sex (Male/Female)	Age (year)	Body Weight (kg)	Creatinine Clearance (mL/min)	
1	Yasuhara (1998)	190	hospitalized MRSA-infected patients	131/59	64.3 ± 13.8 [19.3–89.6]	52.3 ± 9.6 [25.5–75]	77.1 ± 50.9 [6.85–not rereported in the original publication]	[3]
2	Buelga (2005)	215	hematological malignancies	119/96	51.5 ± 15.9	64.7 ± 11.3	89.4 ± 39.2	[14]
3	Staatz (2006)	102	unstable renal function following cardiothoracic surgery	71/31	66 ^b^ [17–87]	74 ^b^ [44–110]	60 ^b^ [12–172]	[15]
4	Yamamoto (2009)	100	adult patients with Gram-positive infections	64/36	65.4 ± 15.1 [25.8–99.7]	52.6 ± 12.7 [28.7–97]	79.6 ± 41.8 [15.3–218.8]	[16]
5	Thomson (2009)	398	adult patients (age ≥ 16)	251/147	66 ^b^ [16–97.0]	72 ^b^ [40–159]	64 ^b^ [12–216]	[17]
6	Dolton (2010)	33	suspected or confirmed serious infection	26/7	72 ^b^ [38–95]	67 ^b^ [48.9–111]	75.0 ± 47.8	[18]
7	Roberts (2011)	206	critically ill patients	127/79	58.1 ± 14.8	74.8 ± 15.8	90.7 ± 60.4 [30–250] ^c^	[19]
8	Purwonugroho (2012)	212	Thai patients (age > 18)	112/100	66.62 ± 18.38	57.64 ± 11.62	35.07 ± 29.83	[20]
9	Adane (2015)	29	extremely obese ^f^	19/10	43 ^b^ [38.5–53] ^d^	147.9 ^b^ [142.8–178.3] ^d^	124.8 ^b^ [106–133.9] ^c,d^	[21]
10	Moore (2016)	14	ECMO ^e^	11/3	47 ± 16 [19–72]	95 ± 27	84 ± 37	[22]
11	Lin (2016)	100	with post-craniotomy meningitis	66/34	51.6 ± 16.9 [18–86]	59.1 ± 10.0 [38–85]	104.7 ± 43.9 [9.5–216.9]	[23]
12	Okada (2018)	75	undergoing allogeneic hematopoietic stem cell transplantation	49/26	49 ^b^ [17–69]	59.4 [39.4–104.5] ^b^	113 [47–253] ^c^	[24]
13	Usman (2018)	144	adult patients (age > 16)	93/51	62 ^b^ [16–88]	79.5 ^b^ [40–177]	89.8 ^b^ [11.3–313.6]	[25]
14	Zhou (2019)	70	geriatric patients with pulmonary infections (age ≥ 65 years)	49/21	78.3 ± 6.96	60.7 ± 10.2	56.3 ± 22.1	[26]
15	Dorajoo (2019)	80	chronic kidney disease	51/29	71.7 ± 13 [31–97]	57.8 ± 15.7 [33.6–103.8]	33.8 ± 10.3 [−60] ^c^	[27]
16	Jaisue (2020)	180	patients with heterogeneous and unstable renal function	102/78	60.8 ± 17.5 [17–97]	54.2 ± 11.7 [30–103]	66.2 ± 56.2 [7.3–281]	[28]
17	Kovacevic (2020)	73	critically ill septic patients	40/33	56.9 ± 17 [20–87]	78.2 ± 14.2 [30–120]	80 ± 44 [14.28–192.9]	[29]
18	Masich (2020)	16	obese with sepsis or septic shock	9/7	62 ^b^ [30–78]	112.7 ^b^ [72.6–129.1]	46 ^b^ [14–123] ^g^	[30]
19	Jalusic (2021)	29	external ventricular drain-associated ventriculitis	14/15	52 ^b^ [44–61] ^d^	80 ^b^ [70–85] ^d^	152 ^b^ [109–174] ^c,d^	[31]

^a^: Values are expressed as mean ± SD [range], ^b^: Value is expressed as median, ^c^: mL/min/1.73 m^2^, ^d^: 25 and 75 percentiles, ^e^: extracorporeal membrane oxygenation therapy, ^f^: body mass index (BMI) ≥ 40 kg/m^2^, ^g^: The calculation of CLcr used the adjusted body weight from Winter et al. [32].

### 2.2. M-Cube Side 2

Virtual patients were generated during the process indicated on side 2 of the M-cube. The simulation generated patient background information and VCM plasma concentrations.

**Figure 3:**
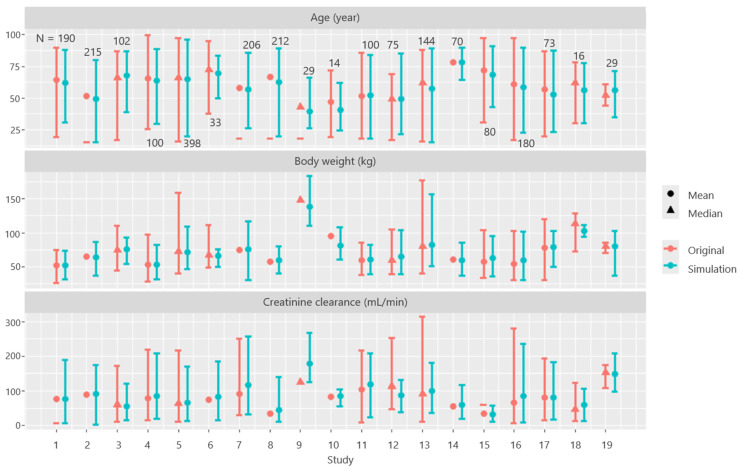
Distribution of patient backgrounds from the original paper reports and simulation-generated patient backgrounds based on these reports. The pink lines represent the original distribution of each data point, directly plotted from the summary statistics (such as mean, standard deviation, minimum, and maximum values) reported in the papers. The blue lines represent the distribution obtained by generating random numbers based on the summary statistics provided in the papers. The numbers shown in the figure represent the number of patients included in each study. The study numbers on the X-axis correspond to the following models: 1: Yasuhara [3], 2: Buelga [14], 3: Staatz [15], 4: Yamamoto [16], 5: Thomson [17], 6: Dolton [18], 7: Roberts [19], 8: Purwonugroho [20], 9: Adane [21], 10: Moore [22], 11: Lin [23], 12: Okada [24], 13: Usman [25], 14: Zhou [26], 15: Dorajoo [27], 16: Jaisue [28], 17: Kovacevic [29], 18: Masich [30], and 19: Jalusic [31]. The study number on the X-axis corresponds to the (study) number in the left column of Table 1. The mean values were broadly consistent between the observed and simulated patients, and the variability was also largely recapitulated, except in some studies that did not report variabilities.

Figure 3 presents the AGE, body weight (BW), and CLcr values obtained from the 19 studies. The first results of the 200 replications were as follows: CLcr (mL/min; mean ± SD; minimum–maximum), 82.26 ± 44.62 (3.3–267); BW (kg), 67.25 ± 18.82 (25.7–183.8). The correlation coefficient calculated using AGE and CLcr data from the 19 models generated by the simulation was −0.23. To assess the reproducibility of the original patient data, the distributions of the generated virtual patient datasets were compared with those of the original dataset. The original data included CLcr of 6.85–313.6 mL/min and BW of 25.5–178.3 kg. The distribution of the virtual patient data adequately covered these ranges (Figure 3). For studies with small sample sizes, it was difficult to reproduce the original data through simulations.

Figure S1 shows the plasma concentration versus time from the initial dose for the 19 models generated using the simulation based on the VCM dosing information from each study. The VCM blood sampling times from six studies [18,19,23,25,27,31] included only trough samples, whereas the remaining 13 studies included blood sampling data at peak or random time points. The integration of 19 studies resulted in 2260 patient cases and 3600 blood sampling points in the results of the first simulation. PopPK modeling was conducted using an integrated dataset of approximately 3600 blood sampling points. Although 200 replication runs generated plasma concentration versus time data, a single representative example is presented.

### 2.3. M-Cube Side 3

PopPK modeling was performed on data integrated from 19 virtual patient models, resulting in 2260 cases. In the covariate investigation, the effects of AGE, BW, CLcr, and the presence or absence of specific diseases or conditions were examined. Based on a likelihood ratio test, CLcr, BW, and provisional PK clusters were identified as statistically significant covariates. No significant associations were found for the other covariates. The final model for CL was:

CLi (L/h) = 2.75 × (CLcr/75)^0.765^ × (BW/65)^0.123^ × (1.881)^provisional PK cluster^ × exp(ηCL) (1)

The term “provisional PK cluster” was used in this study to describe a pharmacokinetically defined group consisting of only sepsis or septic shock [30], hematologic malignancies [14], post-craniotomy meningitis [23], and critically ill patients with sepsis [19]. During covariate exploration, four diseases in which the eta of CL in the base model showed a significantly elevated tendency were incorporated as categorical covariates, defined as a “provisional PK cluster.” In other diseases or models, although CL tended to be approximately 10% higher or lower, these patterns were not statistically significant and therefore were not included in the cluster. The final estimate of 2.75 L/h obtained using the M-cubed method was approximately half that of the Adane model [21] for extremely obese patients and the Lin model [23] for post-craniotomy patients. This was also more than double the estimate obtained from the Dorajoo model [27] for patients with chronic kidney disease.

**Figure 4:**
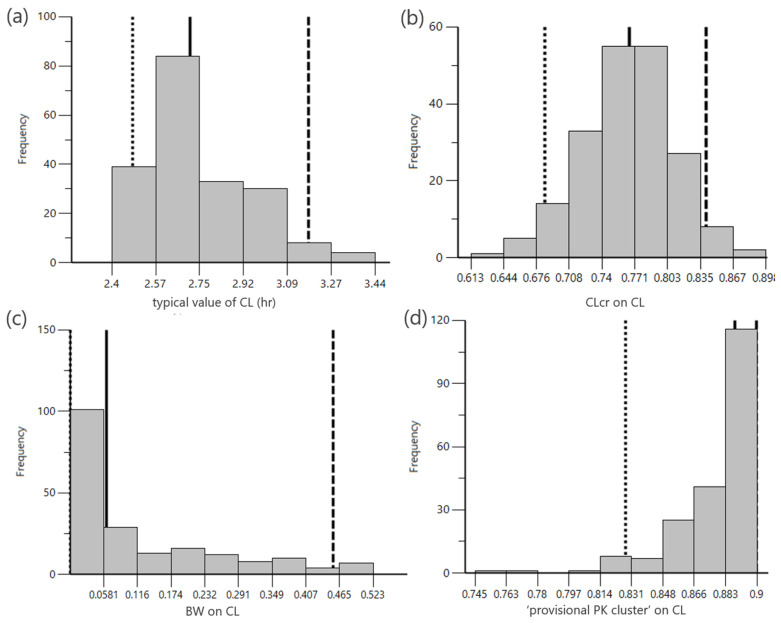
Distribution of population estimates for the final model obtained from 200 simulations. (**a**) typical value of CL, (**b**) the effect of CLcr, (**c**) the effect of BW, (**d**) the effect of “provisional PK cluster.” The term “provisional PK cluster” was used in this study to describe a pharmacokinetically defined group consisting of only sepsis or septic shock, hematologic malignancies, post-craniotomy meningitis, and critically ill patients with sepsis. These four histograms represent the distributions of the four population parameter estimates from the final model, based on 200 simulations. The solid, dashed, and dotted lines represent the median, 95th percentile, and 5th percentiles, respectively.

The final model was replicated 200 times; Figure 4 presents a histogram of the CL values obtained from these 200 iterations. The histogram of CL estimates followed a distribution of 2.75 ± 0.213 (population estimate ± standard error). The typical CL value of the model obtained using the M-cubed method did not differ markedly from the values of each model (Table S1). The goodness-of-fit plots for the final model are shown in Figure S2, and a representative result is presented. The prediction of the VCM concentrations in all patients and diseases was explained using the final model.

## 3. Discussion

A novel methodology for constructing a popPK model that accounts for diverse patient backgrounds was developed by integrating existing models and generating virtual patient data with corresponding drug concentration profiles. In some popPK reports for VCM, the numbers of cases and blood concentration points were inadequate for modeling purposes. This was addressed by combining data from multiple studies. In particular, blood concentrations at clinically difficult time points (e.g., near peak and trough levels), which are frequently missing owing to invasiveness, can be supplemented by data integration. The existence of hundreds of models complicates the selection of the most suitable model for individualized dosing design. This is because most existing models are developed from narrowly selected patient populations. We aimed to reduce the complexity of model selection for individualized dosing by integrating models across a wide range of patient backgrounds. Moreover, VCM dosage adjustment depends on pharmacist expertise. An integrated model may thus help support dosing decisions and reduce the dependence on individual expertise.

To assess the utility of the M-cubed method, VCM was selected as the model drug. Even in VCM models with different diseases, virtual patients can be generated based on representative values from the original literature using the newly developed M-cubed method. The final model had statistically significant covariates for CLcr, BW, and “provisional PK cluster,” and the between-subject variability of CL was 61.56% (45.35%–80.38%) (Figure 4). The fit of the final model was satisfactory. Therefore, it was possible to integrate 19 models that were limited to specific patient populations.

TDM is recommended for VCM in clinical use to ensure efficacy while avoiding the occurrence of adverse effects, such as nephrotoxicity and VIII cranial nerve disorders. Since VCM has been shown to reduce the risk of renal impairment, area under the concentration time curve-guided dosing is recommended [33]. Extensive patient data collected through TDM have been accumulated in clinical settings, resulting in hundreds of reported popPK analysis models constructed using these data (Figure 2). However, the subjects of these individual studies have often been restricted to specific, narrow patient populations, thereby limiting their utility for general clinical application. This situation has led to confusion regarding which PopPK model should be utilized for designing VCM dosing regimens for patients in clinical practice.

Based on the critical need to address this clinical question, and with the conviction that a versatile model applicable to diverse patient characteristics would be highly beneficial in the clinical setting, we conducted the present study. The integrated model derived using the M-cubed method encompasses diverse patient backgrounds originating from multiple studies, suggesting its potential for broad applicability in designing dosage regimens for a wide spectrum of patients in clinical practice.

To the best of our knowledge, only three studies integrating popPK models were identified by Claisse et al. [34]; however, their study focused on patients undergoing renal replacement therapy who were administered VCM. Therefore, we developed a method for integrating popPK models without limiting the data to specific diseases or conditions. Claisse et al. also attributed the large errors in typical values and between-subject variability estimates among the studies to insufficient power due to the small number of cases and sampling points. Although we also included studies with sparse sampling data, further research is required to resolve this lack of power.

Colin et al. [35] provided an approach for pooling reported population analyses; individual raw data were obtained by contacting the original investigators via e-mail. Although their method facilitated the construction of a more precise model, our approach differed in that it aimed to maximize the use of information available only from published literature. The model selection and averaging algorithm proposed by Uster et al. integrates predictions from multiple models based on an existing clinical dataset and virtual patient data generated from published models [36]. In their approach, individual raw data from their own institution were used, and multiple published models were compared against these data. In contrast, we integrated virtual patient data derived from published patient backgrounds with virtual plasma concentration data created from published models to construct a combined model. Our integrated model offers the advantage of being applicable to dosing design for diverse patient populations. Although Uster et al. [36] proposed a method for integrating predictions from published models, the M-cubed method differs in that a model was constructed by integrating information from multiple published models.

Our study focused on models that included only CLcr as a covariate for the CL model. There are four reasons for this. According to the literature review, CLcr is a covariate that can explain most of the individual variability in CL, even in patients with diverse backgrounds such as varying grades of obesity, severe renal impairment, or regarding post-surgical conditions. Although there are reports of models using the eGFR as a covariate, there is no consensus on whether to use CLcr or eGFR for VCM dosing design. The Yasuhara model [3], which used CLcr, was incorporated into representative applications for VCM dosing design in Japan. The model that served as the basis for the present clinical question was developed for a Japanese population [3]. The primary objective of the current study was to propose a novel methodology, and subsequent investigations will be directed toward the application of this methodology to global models. The M-cubed method focused on the simplest model, incorporating only CLcr, to examine the extent of its impact. Furthermore, the model examined in this study was derived from patient data of a Japanese population. Future investigations should expand the scope to include model-informed precision dosing models that have been developed and applied globally.

The summary statistics of the patient data reported in each study were not standardized. Some studies report the mean ± standard deviation, others report the median with the maximum and minimum values, and some report the median or the 25th and 75th percentiles. The quality of the simulated patient data influenced the accuracy of the parameter estimation. The generation of simulated patient data was a crucial process, but it remains a limitation. Furthermore, from the original text and figures, it was not possible to ascertain the relationships between the individual data points (e.g., age, renal function, sex, and BW). Given these issues, there may be a need for guidelines to standardize summary statistics reported in articles related to model analysis.

The patients from the four diseases identified as statistically significant in the final model parameter, “provisional PK cluster,” indicated a potential 1.88-fold increase in CL compared with normal patients (range: 1.77–1.90) [14,19,23,30]. Four clinical conditions, sepsis or septic shock, hematologic malignancies, post-craniotomy meningitis, and critically ill patients with sepsis, were found to substantially increase CL. This finding was attributed to the observation that between-subject variability in CL, based on a model incorporating only CLcr and BW, tended to be above average in patient datasets corresponding to these four conditions. However, because of the use of virtual patient data, we were unable to identify any underlying mechanisms.

Our study has some limitations. The PK parameters in this study focused on CL. Covariates for the central compartment volume of distribution, peripheral compartment volume of distribution, and intercompartmental CL were not examined. The objective of this study was not to identify covariates for all PK parameters but to establish a novel methodology for generating virtual patients from individual popPK studies that only provide representative values and perform modeling. Therefore, covariates of PK parameters other than CL should be explored in future studies. There were also limitations regarding the handling of correlations between covariates in the generation of virtual patients. Potential approaches to addressing this issue include resampling existing datasets and using multivariate distribution models. However, these methods have inherent limitations. Specifically, resampling from existing data is constrained by combinations of covariates present in the original dataset. For example, databases such as NHANES [37], which include mainly healthy adults, may not contain information on whether individuals with specific backgrounds received particular medications. To simulate real-world patient populations more accurately, it may be necessary in the future for individual studies to report more detailed relationships between variables. The model constructed using the M-cubed method encompassed a broad range of patient backgrounds; however, its applicability in real-world clinical practice requires further validation. In the present study, external datasets representing diverse patient populations were not available; therefore, such a validation could not be conducted. Future investigations should aim to evaluate whether this approach can be applied in clinical practice.

Despite these limitations, the integrated model developed using this approach holds promise for clinical applications, such as integration into dosing support software or the development of nomograms. Its implementation may facilitate individualized dosing strategies across diverse patient populations, thereby reducing dependence on the experience of clinical pharmacists.

## 4. Materials and Methods

### 4.1. An Overview of Integrating Multiple popPK Models

The method for integrating multiple popPK models is termed the model-simulated model-based meta-analysis (M-cubed) method. A conceptual diagram of the M-cubed method is shown in Figure 1. The three sides of the cube in Figure 1 represent the three key aspects of the M-cubed method.

Although conventional meta-analyses in clinical research integrate effect sizes derived from different studies (e.g., odds ratios, risk ratios, and mean differences) and data from tables and figures, the M-cubed method proposed in this study is a data-generating approach that extends the methodology of meta-analysis (M-cube side 1). By extending the methodology of meta-analysis and using simulation techniques, our approach facilitated the integration of multiple popPK models across various patient backgrounds. Subsequently, virtual patient data were generated through simulations to replicate patient data from studies representing a wide range of patient backgrounds (M-cube side 2). The generated virtual patient data were integrated to construct a model (M-cube side 3), which was applicable to patient backgrounds from each study. VCM was selected as a model drug to validate the utility of the M-cubed method.

### 4.2. M-Cube Side 1: Selection of popPK Models for Integration

Side 1 of the M-cube represents a collection of popPK studies. Studies on VCM were searched in the PubMed database (https://pubmed.ncbi.nlm.nih.gov/) as of September 10, 2022. The PubMed search formula was [“vancomycin population pharmacokinet*”]. Screening for studies involving VCM modeling was conducted by two authors who reviewed the abstracts and made judgments.

The inclusion criteria for studies were: (1) adult patients (aged 18 years or older) who were administered VCM; (2) popPK model for CL included only CLcr as a covariate; and (3) parametric analysis assuming probability distributions.

The exclusion criteria for studies were: (1) patients under 18 years of age; (2) the VCM model for CL included covariates other than CLcr; and (3) nonparametric analyses that do not assume any probability distributions, as a parametric model is critical in generating virtual patient data.

Of the VCM models selected based on the above criteria, some contained population CL values (L/h) that were considered biologically implausible. To determine whether these values were biologically plausible, we used the fact that, for the renally excreted drug VCM, CLcr may be considered an indicator of renal function. Assuming that VCM is predominantly eliminated through the kidneys, the corresponding CL should be 3 L/h when CLcr is 50 mL/min. Therefore, a CL of 3 L/h was used to represent a typical CL. A CLcr of 50 mL/min was substituted into each popPK model and the population mean CL L/h was calculated for each model. If the calculated population mean CL in each modeling study deviated more than twofold or less than half of the typical CL (>6 or <1.5 L/h), the model was deemed biologically implausible. This verification of the deviation from the typical CL was performed for all models selected according to the inclusion and exclusion criteria. As a result, 19 popPK models were selected. Because this study aimed to integrate models across various patient backgrounds, no disease-specific exclusion criteria were applied.

### 4.3. M-Cube Side 2: Generation of Virtual Patients

Side 2 of the M-cube represents the virtual generation of the study data by generating virtual data through simulation. Virtual patient data used in the development of the PK model of each population were generated as accurately as possible.

#### 4.3.1. Generation of Virtual Patient Background

Simulated virtual patient data were generated for each selected popPK model, with the number of cases matching the sample size of each study.

First, the summary statistics of the characteristics provided in the studies were used to generate patient background data. As data published in journal articles are not presented in their raw form and studies report only aggregated summary statistics (e.g., means and standard deviations), it was not possible to fit a popPK model using only these summarized values. Therefore, virtual patient data were simulated using summary statistics, such as means and standard deviations. Random numbers following a reasonable probability distribution (normal, log-normal, or uniform) were generated using the statistical software R (version 4.2.1, R Foundation for Statistical Computing, Vienna, Austria). To confirm whether the generated random data accurately replicated the patient distribution in the original study, the summary statistics of the generated patient background data were calculated.

For studies in which the unit of CLcr based on the Cockcroft–Gault formula was corrected for body surface area (mL/min/1.73 m^2^), the CLcr was recalculated to the uncorrected unit (mL/min) by adjusting for body surface area [11]. Body surface area was recalculated using the Du Bois formula [38]:

Body surface area (m^2^) = BW^0.425^ × height^0.725^ × 0.007184 (2)

When generating virtual patient data, the correlation between patient background variables was also considered. The correlation coefficient between AGE and CLcr (r = −0.58) was calculated using data from our in-house data of 246 adult patients who were administered VCM. The correlation between BW and CLcr was not directly considered because BW and AGE, as well as serum creatinine and AGE, are correlated; thus, the correlation between AGE and CLcr indirectly accounts for these relationships.

#### 4.3.2. Generation of Vancomycin Plasma Concentration

Virtual sampling data were generated based on information from each popPK study. The sample size matched the number of cases in each study. First, to replicate the patient data, the following information from the relevant literature was used: AGE, BW, CLcr, VCM dosage regimen, dosing interval, infusion duration, blood sampling time points, average number of blood samples per patient, and steady-state conditions. The VCM dosage data were generated in accordance with the information provided in the text of each study, including dose adjustments based on renal function, loading doses, and maintenance. The generation of blood sampling times followed the information provided in the original study. If the original study only described “trough” or “peak,” the sampling time points were classified into three categories: peak, random (from post-peak to pre-trough), and trough. The actual sampling times were generated based on a uniform distribution within each sampling time window. Some studies did not provide specific information regarding the blood sampling time points. In such cases, data were generated under the assumption that for a twice-a-day dosing regimen, blood sampling occurred at the steady-state trough concentration. In reality, drug concentrations cannot fall below zero. However, in this simulation study, large intra-individual variability may have occasionally resulted in negative vancomycin plasma concentrations. Therefore, simulated plasma concentrations below zero were excluded. As the number of negative concentrations varied, the total number of data points differed across simulations. Virtual patient data were generated for each relevant study and combined into a single integrated dataset.

### 4.4. M-Cube Side 3: popPK Modeling of the Integrated Virtual Patients

Side 3 of the M-cube refers to popPK modeling on the integrated patient data generated on side 2 of the M-cube. Similar to the standard analysis, the optimal model was explored through the usual model-building process. A two-compartment model was used as the PK model. A total of 200 replicates were conducted to construct the base model, yielding 200 population parameter estimates, which were then used to calculate the mean and standard error of the 200 estimates. The standard model-building procedure (including covariate model exploration and final model development) was repeated 200 times.

### 4.5. PopPK Model Building

Virtual data were generated using R software version 4.2.3; popPK analysis was conducted using nonlinear mixed-effects modeling program, Phoenix NLME™ software (version 8.4, Certara USA, Inc., Princeton, NJ, USA). The base model was a two-compartment model. Between-subject variability was described using an exponential error model, and residual unidentified variability was described using a proportional error model. We investigated the potential covariates (AGE, BW, CLcr, and diseases) affecting the CL of VCM. Covariate selection was performed using stepwise forward inclusion and backward eliminations. This process was guided by the objective function value (OFV). The change in the OFV reliably follows an χ^2^ distribution. To construct the full model, we added covariates with OFV > 3.84 (*p* < 0.05) in the forward inclusion step. In the backward elimination process, covariates with OFV > 6.63 (*p* > 0.01) were excluded from the full model. A power model was used to describe a continuous covariate normalized to the population median value of the covariate. The formula was:

(3) $$TVi=TVpop\times ({\frac{COVi}{COVmed})}^{\theta }$$

where *TVi* is the typical value representing the individual PK parameter estimates based on the individual covariates, *TVpop* is the typical value of the population mean parameter, *COVi* is an individual’s covariate, *COVmed* is the median of each covariate, and *θ* represents a scale factor related to the effect of the covariate on the PK parameter.

The model for categorical covariates was:

(4) $$TVi=TVpop\times ({1+\theta )}^{COVi}$$

where *COVi* represents an individual categorical covariate that takes the value of 0 or 1. Parameter estimation for the nonlinear mixed-effects model was performed using the first-order conditional estimation–extended least squares method. Goodness-of-fit plots were used for model evaluation.

## 5. Conclusions

TDM has been a predominant method for developing popPK models; however, selecting an appropriate model for clinical use remains challenging because of limited variability in patient data. We tested the feasibility of constructing a unified model from simulated patient data representing diverse patient backgrounds using the M-cubed method. Consequently, this approach is a promising methodology. Using VCM as an example, the M-cubed method integrated data from 19 popPK models, including 2260 cases, to develop a clinically applicable model. The M-cubed method may also be used to integrate various models for a specific drug, particularly in pediatric patients, and may even be extended to pharmacodynamics, potentially facilitating broader model unification. This approach may address the need for individualized dosing strategies for patients with atypical PK profiles in clinical settings.

## Data Availability

The original contributions presented in this study are included in the article/Supplementary Materials. Further inquiries can be directed to the corresponding author.

## Supplementary Materials

The following supporting information can be downloaded at: https://www.mdpi.com/article/10.3390/ph18111748/s1, Figure S1: The relationship between VCM plasma concentration and time elapsed after the initial administration (results for one out of 200 simulations); Figure S2: Goodness-of-fit plots from a single run of the final model (out of 200 simulations); Table S1: Clearance models and their parameters of 19 models.

## Abbreviations

The following abbreviations are used in this manuscript:

BW	Body weight
CLcr	Creatinine clearance
MRSA	Methicillin-resistant *Staphylococcus aureus*
NLME	Nonlinear Mixed Effects Model
PK	Pharmacokinetics
popPK	Population pharmacokinetics
OFV	Objective function value
TDM	Therapeutic drug monitoring
VCM	Vancomycin
