# Supplementary material for: Generating and Modeling Virtual Patient Data from Published Population Pharmacokinetic Analyses: A Vancomycin Case Study
Source: Pharmaceuticals (Basel). 2025 Nov 17;18(11):1748. doi: 10.3390/ph18111748 (PMC12655213; doi:10.3390/ph18111748)
Supplement: Supplementary file 1 [file pharmaceuticals-18-01748-s001.zip › Figures S1 and S2.pptx]

## Slide 1
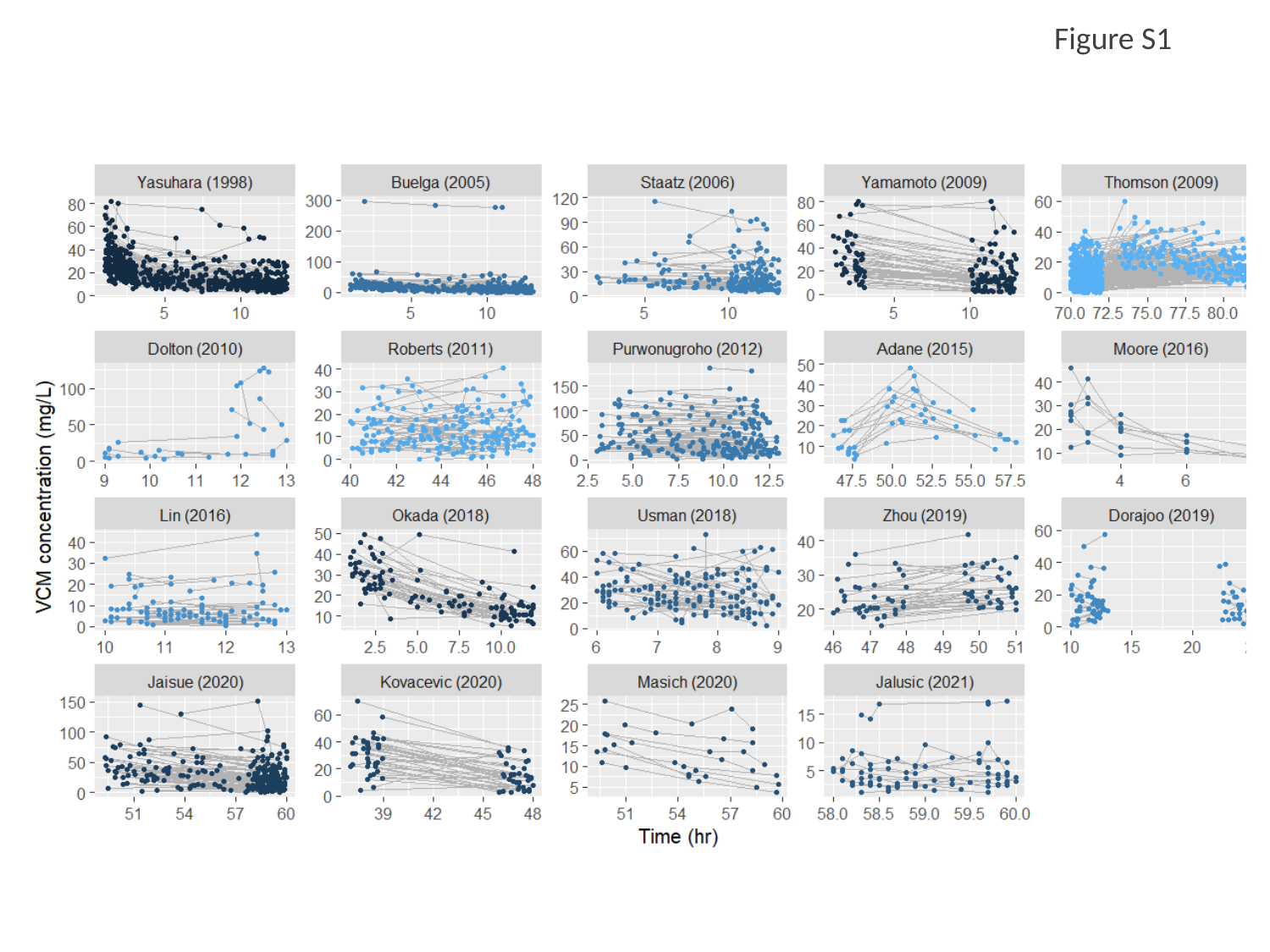

Figure S1

## Slide 2
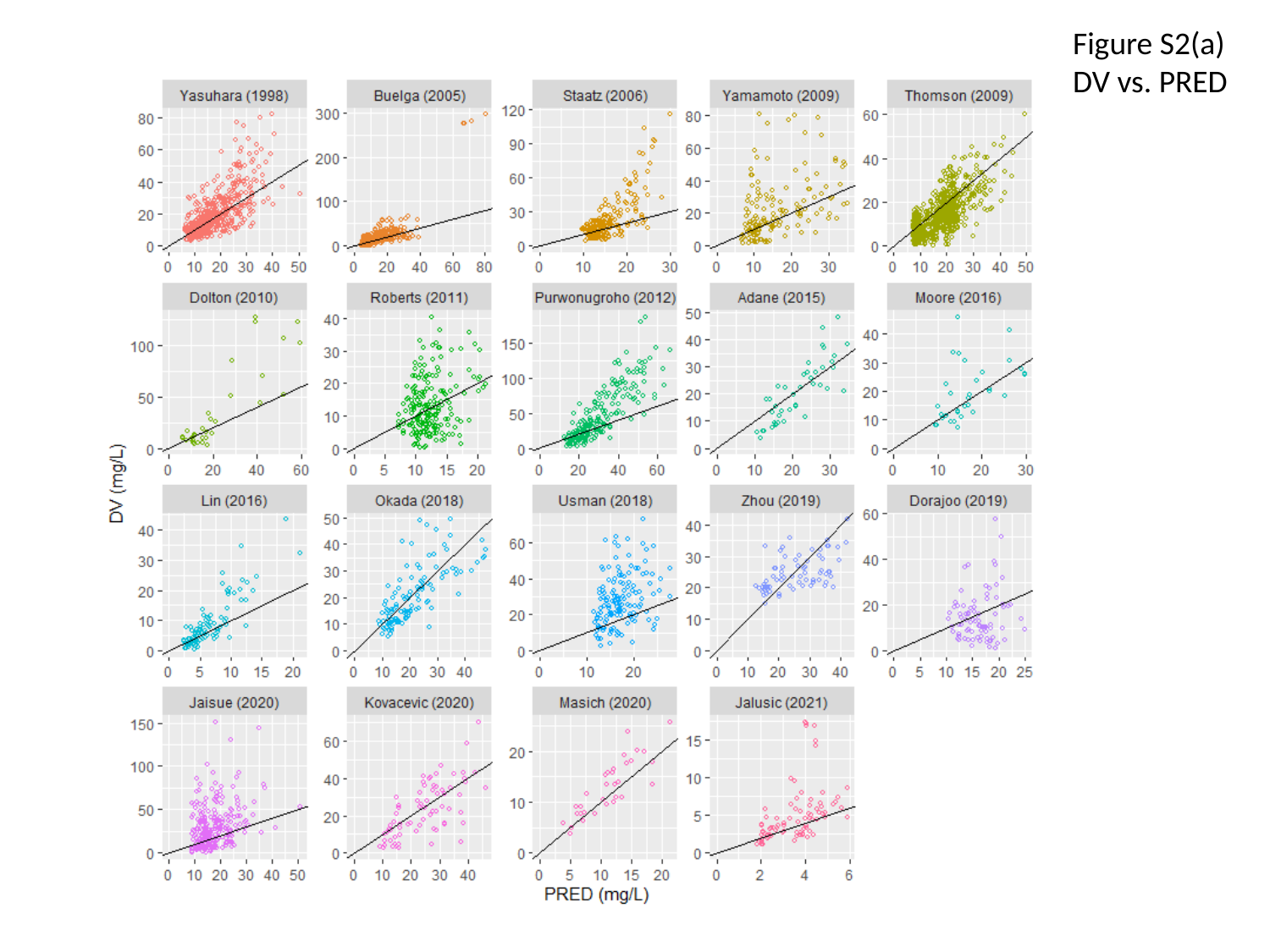

Figure S2(a)
DV vs. PRED

## Slide 3
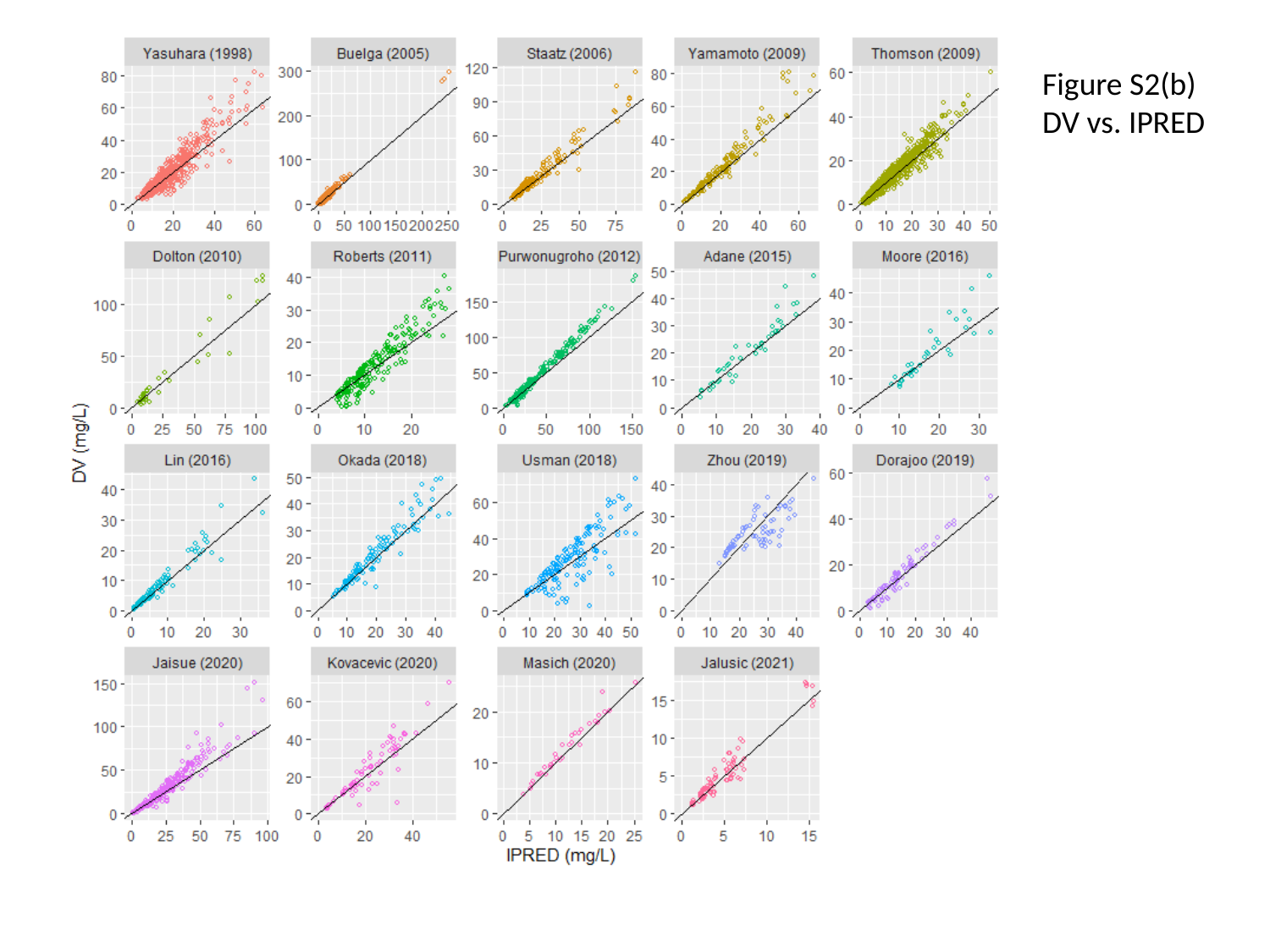

Figure S2(b)
DV vs. IPRED

## Slide 4
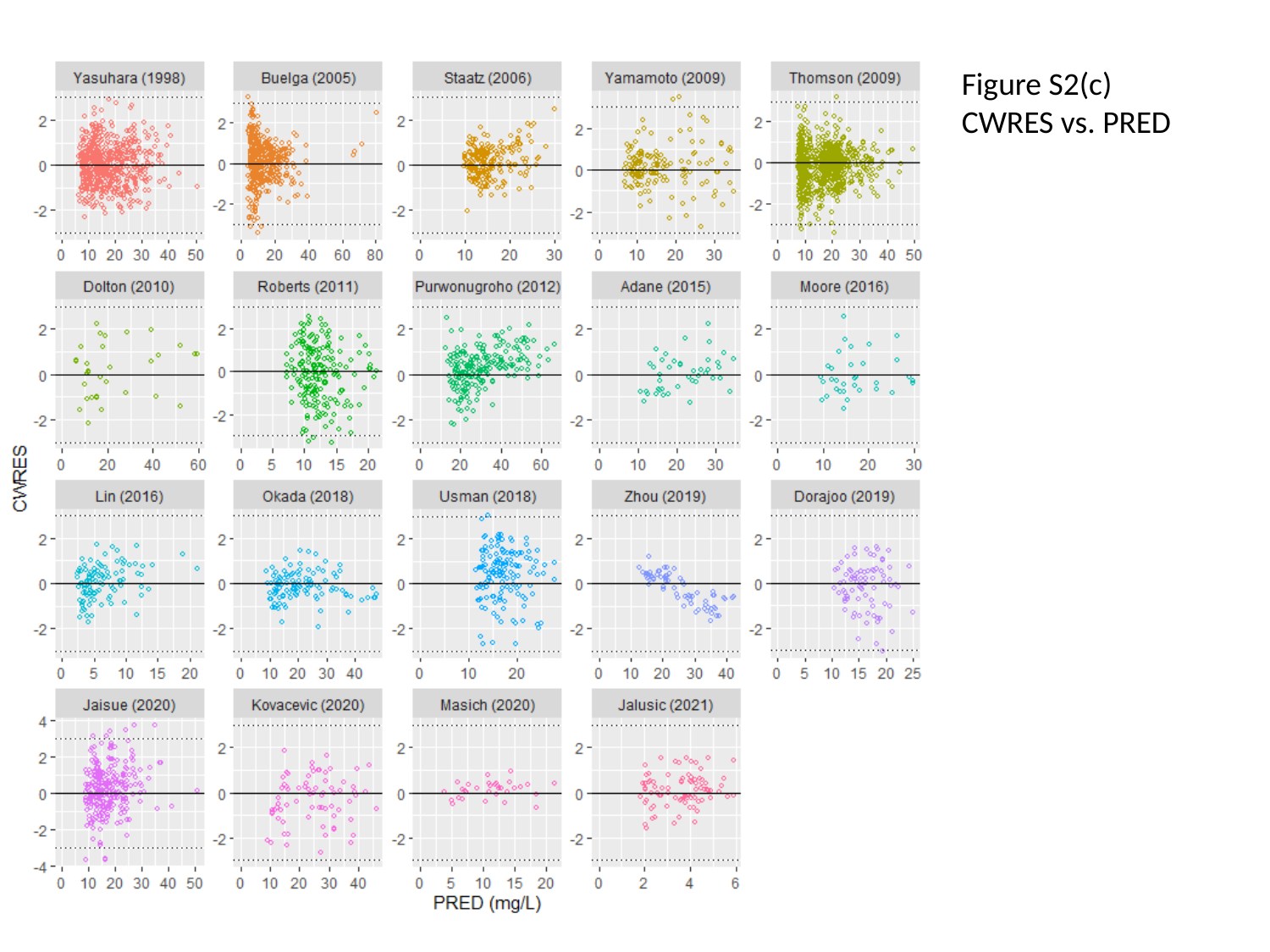

Figure S2(c)
CWRES vs. PRED

## Slide 5
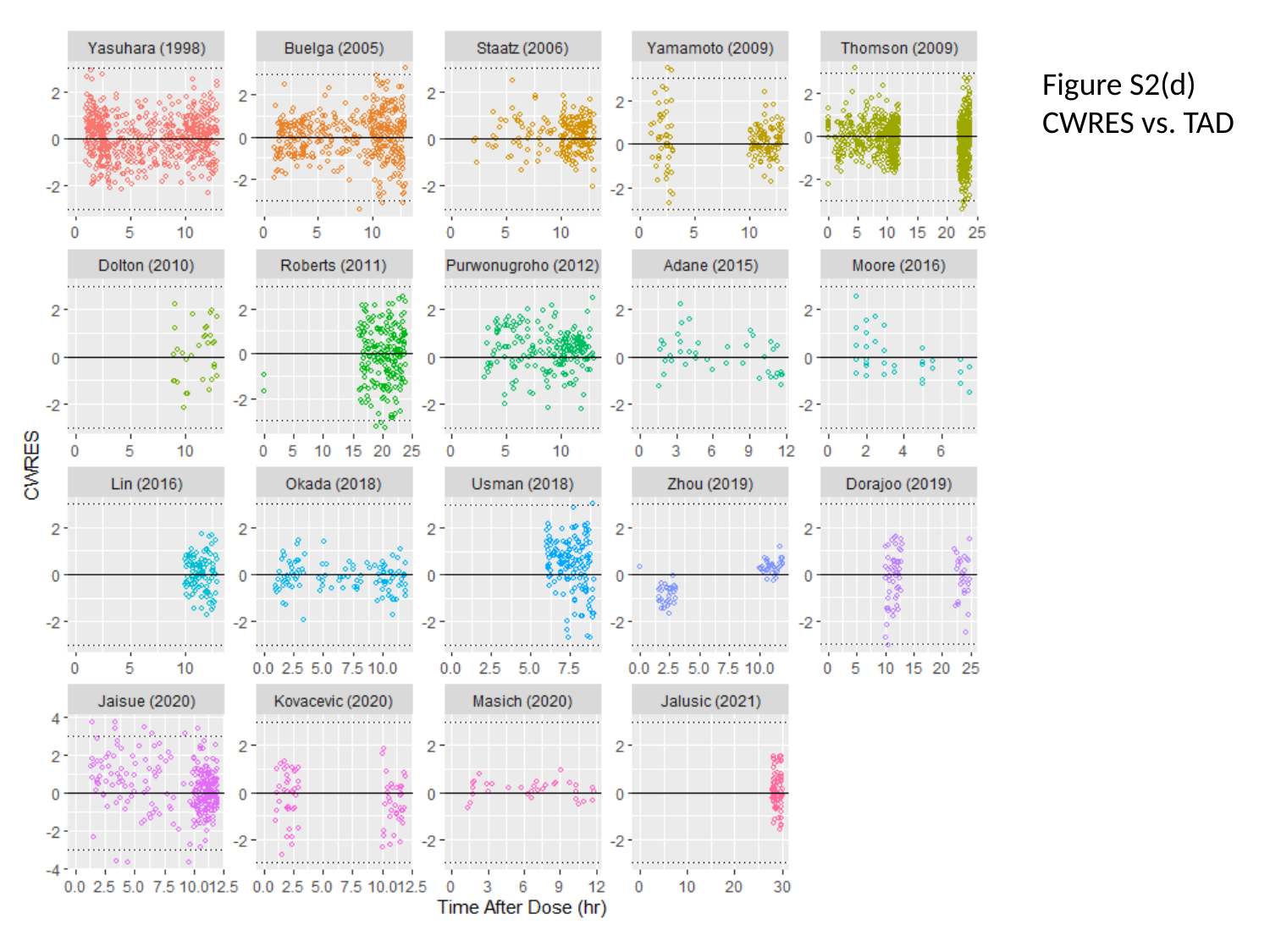

Figure S2(d)
CWRES vs. TAD
