# Supplementary material for: Generating and Modeling Virtual Patient Data from Published Population Pharmacokinetic Analyses: A Vancomycin Case Study
Source: Pharmaceuticals (Basel). 2025 Nov 17;18(11):1748. doi: 10.3390/ph18111748 (PMC12655213; doi:10.3390/ph18111748)
Supplement: Supplementary file 1 [file pharmaceuticals-18-01748-s001.zip › Table S1.pdf]

Table S1. Clearance models and their parameters of 19 models.

study		comp	CL (L/h)			Vd (L)			BSV (%)	RUV		Ref.
no.	1st author (year)		formula	parameter	estimate	formula	parameter	estimate	exp	add (mg/L)	pro (%)	
1	Yasuhara (1998)	2	θ1×CLcr, if CLcr ≤ 85 mL/min	θ1	0.0478	Vss[f]=θ3	θ3	60.7	38.5		23.7[a]	(3)
			θ2, if CLcr > 85 mL/min	θ2	3.51							
2	Buelga (2005)	1	θ1×CLcr	θ1	1.08	Vd=θ2×BW	θ2	0.98	28.16	3.52		(14)
3	Staatz (2006)	1	θ1×(1+θ2×(CLcr-60))	θ1	2.94	Vd=θ2×BW	θ2	1.15	25	1.7	17	(15)
				θ2	0.0209							
4	Yamamoto (2009)	2	θ1×CLcr+θ2, if CLcr < 85 mL/min	θ1	0.0322	V1=θ4×BW<br>V2=θ5	θ4<br>θ5	0.478<br>60.6	37.5		14.3[a]	(16)
				θ2	0.32							
			θ3, if CLcr ≥ 85 mL/min	θ3	3.83							
5	Thomson[b] (2009)	2	θ1+θ2×(CLcr-66)	θ1	2.99	V1=θ3×BW<br>V2=θ4×BW	θ3<br>θ4	0.675<br>0.732	27	1.6	15	(17)
				θ2	0.0154							
6	Dolton[c] (2010)	2	θ1×(CLcr/109)	θ1	4.7	V1=θ2×(BW/70)<br>V2=θ3×(BW/70)	θ2<br>θ3	68.4<br>73	32.7	0.2292	29.30	(18)
7	Roberts[d] (2011)	1	θ1×(CLcr/100)	θ1	4.58	V1=θ2×BW	θ2	1.53	38.9	2.4	19.9	(19)
8	Purwonugroho (2012)	2	θ1×CLcr	θ1	0.044	V1=θ2×AGE<br>V2=θ3	θ2<br>θ3	0.542<br>44.2	35.78	4.51		(20)
9	Adane (2015)	1	θ1×(CLcr/125)	θ1	6.54	Vd=θ2×BW	θ2	0.51	26.7		18.90	(21)

10	Moore (2016)	2	θ1+θ2×(CLcr-84)	θ1	2.83	V1=θ3×(1+θ4×(BW-95))<br>V2=θ5×(1+θ6×(BW-95))	θ3<br>θ4<br>θ5<br>θ6	24.2<br>0.00638<br>32.3<br>0.0169	77		0.0067	(22)
				θ2	0.0154							
11	Lin (2016)	1	$θ1×(CLcr/104.71)^{θ2}$	θ1	7.56	Vd=θ3	θ3	101	31		20.2	(23)
				θ2	0.886							
12	Okada[d] (2018)	2	$θ1×(CLcr/113)^{θ2}$	θ1	4.25	$V1=θ3×(BW/59.4)^{θ4}$<br>V2=θ5	θ3<br>θ4<br>θ5	39.2<br>0.78<br>56.1	25.2		17.2	(24)
				θ2	0.7							
13	Usman (2018)	1	θ1×(1+θ2×(CLcr-89.8))	θ1	2.32	Vd=θ3	θ3	19.2	20.4		38.5	(25)
				θ2	0.0018							
14	Zhou (2019)	1	$θ1×(CLcr/56.28)^{θ2}$	θ1	2.45	Vd=θ3	θ3	154	17.4	0 FIX	6.57	(26)
				θ2	0.542							
15	Dorajoo[d] (2019)	1	θ1+θ2×CLcr	θ1	1.3	Vd=θ3×BW	θ3	1.23	54.4	2.46	0.001	(27)
				θ2	0.023							
16	Jaisue (2020)	2	θ1×CLcr+θ2, if CLcr>120 is =120	θ1	0.021	V1=θ3<br>V2=θ4	θ3<br>θ4	13.8<br>44.7	17.2[e]		17.6[a]	(28)
				θ2	0.111				45[e]			
17	Kovacevic (2020)	1	θ1×CLcr+θ2	θ1	0.024	Vd=θ3×BW	θ3	0.511	56.6		34.5	(29)
				θ2	1.93							
18	Masich (2020)	1	$θ1×(CLcr/46)^{θ2}$	θ1	3.23	Vd=θ3	θ3	85	31.5		8.89	(30)
				θ2	0.69							

19	Jalusic (2021)	3	θ1×(CLcr/152) ^{θ2}	θ1	5.15	V1=θ3<br>V2=θ4	θ3<br>θ4	41.13<br>0.32	34.9	3.16	18.5	(31)
				θ2	0.407							

comp, number of compartments; BSV, Between-subject variability; RUV, residual unidentified variability; exp, exponential error model; add, additional error model; pro, proportional error model, a, exponential error model, b, Thomson's model structure is not included in the study. Based on the annotations in Table suppl 1 and the parameter estimates, we proposed a model structure in which the calculated CL can be interpreted as biologically plausible., c, Dolton's model also reports interoccasion variability, but this was ignored when generating blood concentration in this study., d, creatinine clearance (mL/min/1.73m^2), e, both θ1 and θ2 have BSV, f, the steady state of distribution.
